# Evaluation of the Quality of Football Teaching in Colleges and Universities Based on Artificial Neural Networks

**DOI:** 10.1155/2022/8001252

**Published:** 2022-05-27

**Authors:** Rui Yang, Hui Lin

**Affiliations:** ^1^School of Physical Education and Health, Hubei University of Traditional Chinese Medicine, Wuhan, Hubei, China; ^2^College of Physical Education and Health, Wenzhou University, Wenzhou, Zhejiang, China

## Abstract

Every country is developing under the concept of artificial intelligence. Many countries are already working on student monitoring systems that allow them to control the student's mentality and analyze each student's behavior with the help of a wireless headband. There is certain high-tech education within the country in a maximum number of schools, which can be considered robotic monetization. With the help of tiny robots inside the classrooms, each student's activeness and engagement level in the classes are captured and submitted to the teacher. All these practical applications help us imagine that there will be a massive response to artificial intelligence in the future of this world. On the other hand, sports management is a critical issue to consider for the country's growth. This research evaluates the quality of football teaching by implementing an Artificial Neural Network model for online mode of education. The proposed model functions with the implementation of Association Rule Mining (ARM) in the intelligent system to monitor the activities of the player by training with the Artificial Neural Network (ANN). The proposed model is compared with the existing K-Mean algorithm, and it is observed that the proposed model has achieved an accurate evaluation of 99.6%.

## 1. Introduction

Nowadays, college football instruction still focuses on discovering universal educational principles rather than considering the educational value of exploring context. There is no denying that this goes against the grain of educational research. Consequently, schools and colleges often neglect the significance of self-organization in football training in favor of placing too much reliance on the effectiveness of other organizations [1]. Self-organization and other organizations are the only ways to accurately evaluate a company's performance in the long run. This results in a paradox: teaching resources cannot be adequately utilized since students are placed in a position of dominance over their own creative and subjective initiative and self-organization [2]. These days, the majority of colleges and institutions' football education programs seldom make use of materials available outside of the classroom. Science and technology advance at a quick pace, and society continues to evolve steadily, so do the educational materials available to teachers [3]. Teaching resources may only be optimized if they are used to their fullest extent. Despite these inconsistencies, football instruction in colleges and universities has been badly hindered by them and has been a key reason why colleges have failed to cultivate talented individuals with great comprehensive skills and high levels of proficiency in the sport. In the current stage of college physical education reform, they have become a paradox that has to be addressed immediately [4].

Football instruction at the college level is just as much of a self-organizing system as the rest of the education system. It is the primary focus of self-organization theory to study the establishment and growth of a complex self-organizing system [5]. Today's rapidly changing social expectations for sports education are not compatible with old football teaching methods and concepts. Individual variations among pupils are sometimes overlooked in football instruction. Many football instructors are struggling with the quality of their instruction [6]. Multiple intelligences are a challenge to instructors' skills in the classroom because of this educational idea. Teachers will be motivated to enhance their skills and knowledge, which will lead to an overall increase in the teaching staff's quality [7]. Football teaching in college sports majors should at least comprise teaching ideas, teaching objectives, teaching material, teaching techniques, and teaching assessment [8]. There should be a focus on democratization, individualization, and diversity in the transition of teaching conceptions. For students' overall development, the teaching aim should be focused on fundamental knowledge and skills training as well as the goals of health promotion, pursuit of practicality, sustainability, and satisfaction of individual requirements, as well as the expansion of the teaching goal of multiple intelligences [9]. The PSO algorithm is used as an example, and the method's optimization process is explained thoroughly in this article, along with a flowchart and stages for the computation [10]. The new and improved PSO algorithm is based on biological phenomena and regulations, in addition to the dynamic multispecies particle swarm approach using the food chain mechanism. In addition, this suggests a food chain and reproduction process, as well as measures to improve the algorithm [11]. When compared to the standard PSO method, the improved technique beats it in terms of optimization ability and optimization effect, making it more difficult to find a local optimum. It has also made a significant difference in the handling of multipeak situations [12]. As a result, when it comes to teaching football, many instructors fail to take into account the impact that nonphysical and nonintellectual aspects have on students' ability to remain physically active throughout their lives. Teachers of physical education should experiment with novel assessment techniques and measurements when the overall nation is so enthusiastic about college football [13].

To establish a “lifelong sports society,” school physical education in China has taken the top priority of training and developing young students' “lifelong sports.” Many issues remain in China's “school sports program,” though [14]. The question of how to improve kids' lifetime sports awareness and ideas via a specific sport has thus become one of the most significant study subjects of our day. Football's ever-increasing popularity has raised the bar for what spectators expect to see on the field [15]. As a result, football players and coaches must constantly enhance their own skills and adopt a more scientific and methodical approach to training in order to succeed. Football players' technological advancements are getting more extensive as time goes on. Physical fitness is necessary for players to be able to adapt quickly to changing game situations on the field [16]. Traditional football players rely mostly on speed or strength training in their training regimens. The player's physical function may be damaged by long-term continuous training in one element, and the increase in their performance may not be the most efficient aim [17]. For practical operation application disciplines, computer platforms are now widely used in different institutions. For this reason, many science and technology colleges have set up multimedia computer rooms for the instruction of students. For this instructional mode to work, the software and hardware must be in place [18].

Research into how to enhance the theoretical research and apply it to athletes' training assistance has become a frontier issue that urgently needs to be studied and developed. Football Training Technology (FTT) necessitates the development of an intelligent integrated system [19]. The Football Network and other associated technologies may be used to create an intelligent FTT system for football players that can handle their personal information as well as the training materials of their coaches. “Football training may help athletes' physical and mental well-being, as well as their interpersonal relationships with their coaches and family [20]. According to the Barquero-Ruiz C report, several young football players left the well-run squad. Barquero-Ruiz C recruited 20 youngsters under the age of 11 and two coaches from 17 different teams in order to validate their findings [21]. Performance assessment instruments, amusement measures, and physical activity scales are all used in conjunction with players and coaches in this multimethod study. Data was gathered via the use of two focus groups [22]. There must be an intelligent approach to organized youth football instruction based on the FTT system that takes into consideration the reasons why some young players leave the football team: overemphasizing technical execution, poor goal defense success rate, and lack of player autonomy and motivation. Low citations and a lack of experimentation were found in the study [23]. The researcher aims to investigate the decision-making and execution of football and to examine the consequences of a complete teaching plan in order to better understand the sport [24]. For the sake of understanding the concept, intervention techniques were used in 21 training sessions based on teaching games that incorporated modifications such as asking questions in the postgame setting. Members of the test bunch, when contrasted with those in the benchmark group, showed further developed direction and execution capacities in passing and spilling after the use of the mediation approach [25]. These discoveries propose that a keen football preparing framework change ought to be thought about to upgrade the preparation and strategies of football players. With no training data, we must resort to a more sophisticated approach to data analysis. The method's complexity prevents it from being widely used in practice [26].

Currently, at China's colleges and universities, computers have been employed to aid in teaching, although the primary manner of instruction remains largely unchanged. According to the results of a study, the majority of multimedia teaching modes are still based on the use of projection equipment and slide teaching materials. Preparation of projection equipment, which is expensive for the school to acquire, is necessary for this method. With the typical chalkboard book, some instructors have difficulties preparing lessons, and the teaching impact is unequal. Also, many schools utilize a screen-sharing climate for education. In any case, study results show that this strategy fills a single need somewhat [27]. One of the developments in this paper is a support vector machine model for activity recognition, which is useful for players to prepare. The general engineering of the FTT-wise coordinated framework was planned; football education was led involving the specific planning of the keen incorporated framework for preparing; and the human-PC connection point of interaction and primary specialized design were intended for use.

An ANN is an algorithm based on a biological neural system model that was built by simulating artificial intelligence. It has become more commonplace as a result of advances in science and technology. An artificial neural network model (ANNM) is a kind of algorithm model used for these neural networks. The growth of a “fuzzy” neural network has been a worry in several models of neural networks [28]. Complex functional links between enormous datasets are often analyzed using neural networks, which entail a number of processes, including storing information in the weight coefficient [29]. It is possible to learn the weight coefficient from input and output samples that are automated and stored in a distributed manner. Furthermore, because of the enormous number of neurons in the neural network algorithm, the whole system can only operate once a significant amount of computation has been performed [30]. The properties of a fuzzy system are diverse. In a fuzzy system, information may be summarized and communicated in a way that is easier to comprehend. The number of rules in a fuzzy system can be regulated and changed to a smaller number than in a neural network computation [31]. However, the acquisition of regulations is expensive and complicated since they are offered or produced by professionals. The two are combined to build a mutant fuzzy neural network, which has both benefits and drawbacks based on its strengths and weaknesses [32]. In dealing with large-scale data analysis issues, this model will perform very well and has a lot of room for growth and expansion. This study focused on evaluation of the quality of football teaching in colleges and universities based on artificial neural networks.

### 1.1. Motivation of the Study

There are two interrelated components to the proposed football teaching in colleges and universities' viewpoint on the issue, the resulting effects and the analysis system. As shown below, a good position estimate from the first segment can be used to deduce the player's posture technique from video footage. A storage-time ANN machine study appears to be its second component. There's a viewpoint series on football instruction at colleges and universities where it is identified and separated from fragmentation. Some other methods appear to offer an additional method for raising accuracy while quickly correcting minor errors in comparison to standard training. Players can use this technique to fix technical problems, develop muscle memory, and improve their abilities. It is a huge opportunity and a huge challenge for higher education when AI techniques and implementations become more widely available. Higher education in sports must deal with both challenges and opportunities as machine learning techniques become more commonplace. To take advantage of machine learning's many advantages, educators must deal with a number of challenges, including the need to establish appropriate standards and provide adequate guidance.

## 2. Materials and Methods

Most commonly, if a person wants to buy a mobile phone, his target will not be only the mobile phone, as it requires a back cover and a tempered glass to cover the front surface. In such a case, even the player would forget about the other products some of the e-commerce sites would auto-recommend and figure out such products to the player when he buys a mobile phone. Here, these kinds of suggestions are made only by the marketing members. Still, when it comes to online e-commerce applications and web pages, there would be a robust algorithm giving the right suggestion for the players instead of a human. All kinds of data are collected and stored within a database, and with the help of Association Rule Mining, it has been one of the cooccurrence patterns. While relating a person's activities, the machine would have it as an “If” and “Then” concept, where if the statement is related as the antecedent, and the second opinion is considered as the consequent, both the functions are performed to understand the players' activity under the Association Rule Mining (ARM).

**Figure 1:**
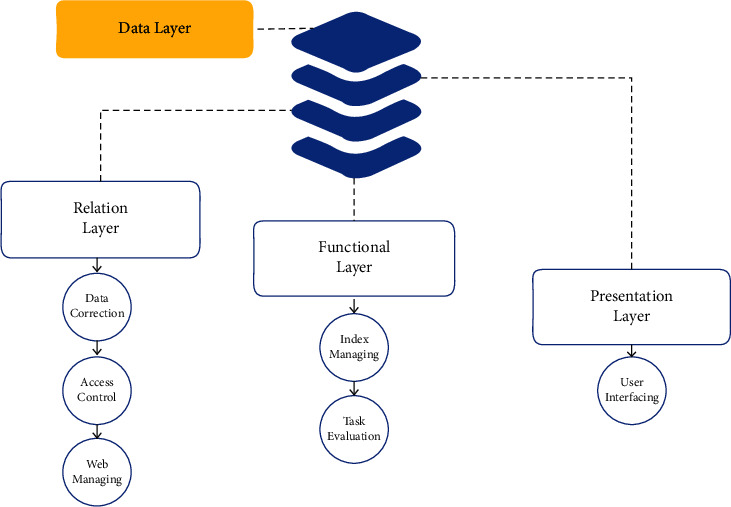
Layer separation under teaching control.

**Figure 2:**
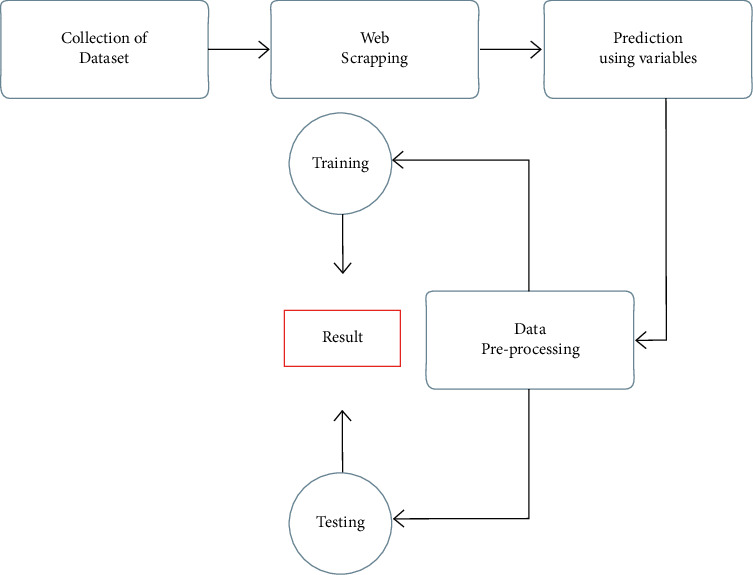
Processes involved in the football teaching.

While getting into the sports session, more than half the students are getting out from their sports due to imbalanced weight and body control. This could be one of the main reasons for the fall of sports management or the availability of the students under sports and games. According to the games, the rules and their regulations would change. In such a case, body maintenance will also have some changes on the basics of games. Moreover, the entire country of China has been updated to nearly 90% of schools and universities to manage their teaching level using computer-based teaching. Still, the mode of regular teaching is the same as the blackboard teaching. At the same time, the other education would relate to music and sports teaching. While if the schools and colleges are trying to teach their students with the help of projection slides, then it would cost an average amount because there should be a projection to overcome the slide teaching for each class. Simultaneously, using the blackboard method requires the teachers prepare more concepts that take enough time to complete their preparation. To avoid all these problems, if there would be a method to find a solution, it means utilizing the computer-based education system for every school and university. By creating a support vector modeling machine, the author explains how the device is used to guide a player before and after completing his practice and test session. FTT intelligent system acts as the essential tool for football teaching. Most few integration systems are still being considered human-computer interaction and interfacing sessions. It usually is more complicated to relate all the player's inequality according to their health conditions and behavior they would differ, and here the Artificial Neural Network helps us by overcoming all such behavior and activities of the player and conveying the result as output, which is represented in Figure 1.  Figure 2 represents the phases of processes involved in analysis of teaching football.

The study assesses the use of machine learning with in serious assessment of football teaching statistics at universities and colleges, employing research procedures including such science articles, audio/video analysis, research methods, or mathematical statistics. The variance among statistical traits throughout all frames surrounding that one, Δ_*i*_, was employed as the characteristic in first step, which is also represented as

(1) $$\begin{array}{c} \sum_{i=1}^{m}\Delta_{i},m_{i}\in m,m_{i}\neq u_{i}^{*}:d_{i}\left(m_{i}^{*},m_{-i}^{*}\right)>i=1\sum_{i=1}^{m}d_{i}\left(m_{i},m_{-1}^{*}\right). \end{array}$$

Also, each participant can learn about their utility in various modes  *d*_*i*_(*n*_1_, *n*_2_,…*n*_*m*_) of strategic approach. A Nash equilibrium position is a profile of {*m*_1_^*∗*^, *m*_2_^*∗*^,…, *m*_*m*_^*∗*^} ∈ *m* strategy that is applied when no change in tactics by the player gives rise to high, low, on the score. Expansion of a large number of players is defined as *Q*=(*Q*_1_, *Q*_2_,…*Q*_*n*_) and also the number of execution using time steps to forecast each task and every source is shown by the equation *P*_*ij*_.

Assume that *D*_*n*_ is indeed the total number of job bidding battles again for the price of one resource at a given time is defined as a resource, and the following equation is produced.

(2) $$\begin{array}{c} Q_{h}=\frac{\sum_{i=0}^{P}\left|D_{n}\left(i\right)-D_{n-1}\left(i\right)\right|}{\text{Width.Heigth}}\;>W_{y}\;P_{ij}. \end{array}$$

*P* is the quantity of the histogram equalization, and  *Q*_*n* _ and *Q*_*n*−1_ are the color histogram equalization of segments *n*with*n* − 1, correspondingly. In equation (2), Height and Width represents the number of pixels in each frame, and *W*_*y*_ represents the thresholds for recognizing one unique particular measure inside a series of Δ_*i*_ deformation levels.

(3) $$\begin{array}{c} A_{n}=\sum_{i=1}^{n-1}\frac{CQ_{n-1}}{Ci_{n-1}}\;\text{for}I-\text{frame.} \end{array}$$

*CQ*, *Ck*and*Ci*, are indeed the CNs for intracoded, forward forecasted, return straight predicted, and also bidirectionally anticipated frames, but *n* is the structural size is represented in equation (3).

(4) $$\begin{array}{c} A_{n}=\sum_{n=1}\frac{IP_{n}}{CN_{n}}\;\text{for}b\text{frame.} \end{array}$$

Humans changed A by (1) erecting a b-frame. Whenever the limit occurs at I-frames, a conception to have more exact results equation (4) is used.

(5) $$\begin{array}{c} A_{n}=\sum_{n=1}^{i}\frac{\left|CQ_{n}-Ck_{n}\right|}{Ci_{n}}\;\text{for}H\text{frame.} \end{array}$$

In equation (5), if *Ck*_*n*_ and *CQ*_*n*_ are both much bigger than Bin, the H-frame RFD presentation is modified to reduce false alerts. Consider the frame evolution depicted in the following equations (6)–(9).

(6) $$\begin{array}{c} \;Q_{n}\left(0\right)=\sum_{n-1}^{n}if\left(Ck_{n-1}-CN_{n-1}\right)>0, \end{array}$$

(7) $$\begin{array}{c} Q_{n}\left(1\right)=\sum_{n=1}^{n}if\left(Ck_{n}-CN_{n}\right)\times \sum_{n=1}^{n}\left(Ck_{n-1}-CN_{n-1}\right)\leq 0\text{ for }b\text{ frame,} \end{array}$$

(8) $$\begin{array}{c} Q_{n}\left(0\right)=\sum_{n-1}^{n}if\left(Ck_{n}-CN_{n}\right)\times \sum_{n=1}\left(Ck_{n-1}-CN_{n-1}\right)>0\text{ for }H\text{ frame,} \end{array}$$

(9) $$\begin{array}{c} m_{i}=\sum_{i=1}^{b}b_{1}m_{i}+b_{2}y_{i}+b_{3}. \end{array}$$

*Qs*_*i* _and*Qy*_*i*_ also are the resampling components for a certain structural element given in the following equation:

(10) $$\begin{array}{c} Qy_{i}=\sum_{i=1}^{b}b_{4}m_{i}+b_{5}y_{i}+b_{6}. \end{array}$$

The interpolation linear parameters of *b*_*i*_′*s* are represented by *m*_*i*_and*y*_*i* _ and appear to be the spots risk prediction of the CB's centroid. The row vector *h*_*i*_=(*m*_*i*_, *y*_*i*_, 1) is described. The coordinates matrix C is subsequently constructed for all blocks, which are not tagged as outlier by vertically appending the row vectors *h*_*i* _. W is a *Q*  × 3 matrix because *M* is the quantity of macro blocks that are not identified as outliers. The vectors *d*_*m*_and*d*_*y*_ are produced by summing all of the *m*_*i*_with*y*_*i* _ for the HBs, which are not identified as outliers. Finally, all linear interpolated parameters *P*_*m*_=(*P*_1_, *P*_2_, *P*_3_^*T*^)with*P*_*y*_=(*P*_4_, *P*_5_, *P*_6_)^*T*^ are combined. Based on such equations, we may write *D*_*s*_=*WP*_*s*_and*D*_*y*_=*WP*_*y*_, which are then calculated for *P*_*x*_and*P*_*y*_, that are utilized in *R*'s form matrix using equation (11) and equation (12).

(11) $$\begin{array}{c} \begin{array}{c} P_{m}=\sum_{m=1}^{T}\left(W^{T}W^{-1}\right)W^{T}D_{m} \end{array}, \end{array}$$

(12) $$\begin{array}{c} P_{y}=\sum_{m=1}^{y}\left(W^{T}W^{-1}\right)W^{T}D_{y}. \end{array}$$

Camera's horizontal movement (CHM) follows the following equation:

(13) $$\begin{array}{c} \text{CHM}=\frac{\sum_{i=0}^{m}P_{3i}}{W}+\int {\left(W^{T}W\right)}^{-1}W^{T}D_{y}. \end{array}$$

Camera's vertical movement (CVM) is specified by the following equation:

(14) $$\begin{array}{c} \text{CVM}=\frac{\sum_{i=0}^{m}P_{6i}}{W}-\int {\left(W^{T}W\right)}^{-1}W^{T}D_{y}. \end{array}$$

Camera's Zoom (CZD): the characteristics listed above will be specified in the following equation:

(15) $$\begin{array}{c} \text{CZD}=\sum_{i=0}^{m}\frac{P_{1i}+P_{5i}}{2}+\int {\left(W^{T}W\right)}^{-1}W^{T}D_{y}. \end{array}$$

*f* reflects the total number of pictures in the image that influenced the outcome in the following equation:

(16) $$\begin{array}{c} \text{Frequency}\left(f\right)=\sum 6495\times \log_{10}\left(1+\frac{f}{900}\right). \end{array}$$

The Frequency (*f*) is the normal frequency scale's measurement unit for sports risk prediction. For risk prediction, the highest proportion has a frequency band of 0–30130 Hz with a fixed frequency spacing. In the following equation, risk prediction is calculated.

(17) $$\begin{array}{c} m_{n}=\sqrt{\frac{2}{k}}\sum_{k=1}^{k}\left(\log m_{k}\;\right)\sum_{n=1}^{n}sin\left[n\left(k-0.6\right)\frac{\pi }{k}\right],n=1,2,\ldots .m. \end{array}$$

Its energy quantifies the variations in speech signals. Linear vitality of recorded conversations *m*_*n*_, where *n*=1,  2,…,  *m*, determined a spectral analysis as given in equation (18).

(18) $$\begin{array}{c} d=\log\sum_{k=1}^{k}m_{n}^{2}+\sum_{n=1}^{n}sin\left[n\left(k-0.6\right)\right]. \end{array}$$

## 3. Result and Discussion

**Figure 3:**
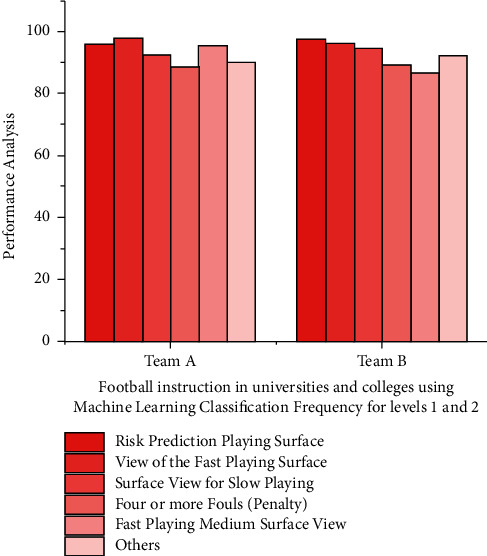
Football instruction in universities and colleges using machine learning classification frequency for levels 1 and 2.

The variance among statistical traits across all frames surrounding that one was used as the characteristic in the first step, which is also shown in Figure 3 that demonstrates how ANN technique must execute information categorization of different data types, filtration, but also remove errors when paired also with judgments feature within this process; furthermore, it incorporates the characteristics of various types of into risk prediction. Components of many kinds are depicted, with data being more distinguished.

Furthermore, the percentage of machine learning models utilized in the study of the various football instructions for Colleges and Universities instruction arrays differs by kind. The increasing expansion of online offerings classified as machine learning has aroused a huge interest regarding.

Machine learning is being used to predict football risks. There appear to be significant discrepancies in classification frequency for team A and team B categories among both university education results and all those reported with in Machine learning collective. Machine learning algorithms share several key traits (see Table 1): the first is that it is democratic; the second is that it is based on such an undercurrent attitude; and thirdly, in regarding education, they would be involved.

**Figure 4:**
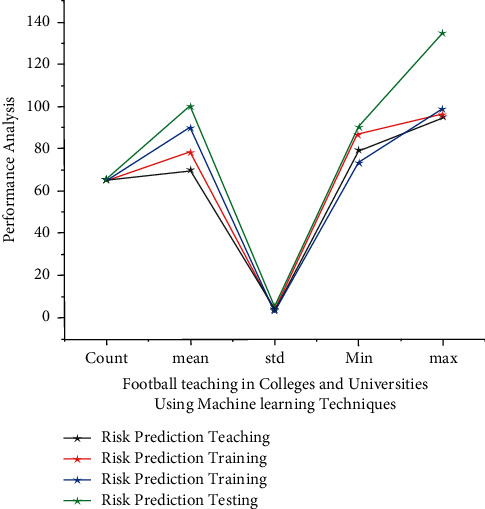
Analysis for football teaching in colleges and universities using machine learning techniques.

A Nash equilibrium position is a profile of {*m*_1_^*∗*^, *m*_2_^*∗*^,……, *m*_*m*_^*∗*^} ∈ *m* strategy when no change in tactics by the player gives rise to high, low, and so on. Expansion of a large number of other players based on this to retrieve in Figure 4 depicts that its variables have a larger influence on the athletic training time, in addition to integrating a few neural network (Machine learning) analysis methodologies, but also selecting high-resolution components to create a good link between identification and risk prediction tracking and different dynamic performances. Sports have been found to have low resolution difficulties during training and assessment. They conducted a number of experimental studies to finalize the multifunctional execution of various knowledge and statistics (refer Table 2).

**Figure 5:**
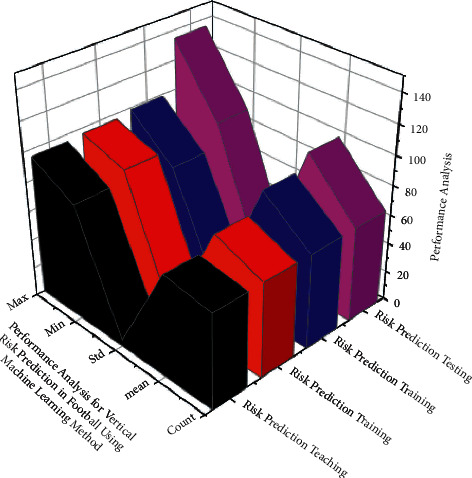
ANN algorithms with analysis for predicting vertical risk in football utilizing machine learning method.

The execution of ANN algorithms in sport events was discovered. Here, *P* signifies the quantity of the color histogram, and  *Q*_*n* _ and *Q*_*n*−1_ denote the histograms of clips *n*with*n* − 1, respectively, as well as the width. The number of pixels in each frame is represented by height, and the thresholds for recognizing single maximal activity in *K*_*h* _ deformation values are represented by *W*_*y*_  based on this, retrieved in Figure 5, but it was found that qualitative distinction conceptual technique is presented purely on machine learning techniques and has been used for processing data with having trained data analysis connection of football teaching through sport education activities but was discovered that this method has seemed to have little more benefits. Machine learning adds to this approach the removal of a financial intermediary's substantial function, which was previously a required feature of the original design (refer to Table 3).

**Figure 6:**
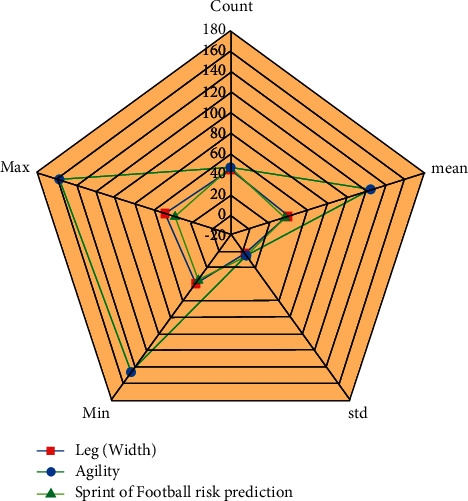
The ANN algorithm in machine learning method employs analysis for leg (width) with agility after which sprinter was used in football teaching in colleges and universities.

The row vector *h*_*i*_=(*m*_*i*_, *y*_*i*_, 1) is described. The coordinates matrix *C* is subsequently constructed for which they are not tagged in outlier by vertically appending the row vectors *h*_*i* _. W is a *Q*  × 3 matrix because *M* is the quantity of macro blocks that are not identified as outliers. The vectors *d*_*m*_and*d*_*y*_ are produced by summing all of the *m*_*i*_with*y*_*i* _ for the HBs, which are not identified as outliers determined in Figure 6 depicting an attempt to introduce the approach of football instruction in colleges and universities players readiness to acknowledge different kinds of training, and also the particle swarm methodology for optimization evaluation and also an ANN algorithm based exclusively on random of sampling matrix. The suggested algorithm can help with the one considered as a predictor of sports participants' pedestrian detection while flowing smoothly; however, at the sacrifice of select accuracy rate, considering numerous learning institutions with machine learning, as well as several potential issues, they also can address how we could make the transition among apparently disparate spheres. It shall concentrate on learning design as an example of how education might have to change, but also other crossing techniques, like the development of relevant technology, would undoubtedly being needed (shown in Table 4).

**Figure 7:**
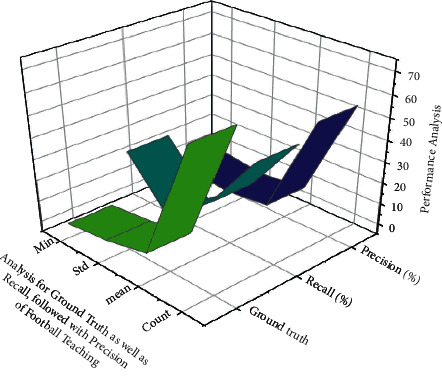
In the machine learning method, the ANN algorithm is used to predict football risk using overall performance analysis.

Finally, all linear interpolated parameters *P*_*m*_=(*P*_1_, *P*_2_, *P*_3_)^*T*^with*P*_*y*_=(*P*_4_, *P*_5_, *P*_6_)^*T*^ are combined. Based on such equations, people may write  *D*_*s*_=*WP*_*s*_and*D*_*y*_=*WP*_*y*_, which are then calculated for *P*_*x*_and*P*_*y*_ using R's form matrix based on this to show the results in Figure 7 suggesting that its method could well tackle the problem of pedestrian identification through fluid movement in sports football education in colleges and universities. The scientists created a modified approach to detect deflection gait zones on ant colonies, as well as ANN algorithms, to improve recognition performance. It is of high quality and can be used to identify a football teaching in higher education institutions taking a step during practice. This can be used to calculate the rotary motion of the foot joints as well as the training effectiveness.

Quality of machine learning: almost all of the discussion about Machine Learning among educators revolves on excellence and also how to maintain it. A collection that allows users to exchange and acquire knowledge may assist in resolving this issue by providing an instructional framework based of resources that can then be updated. Operators would be able to see who generated each learning design, although some programmers will be regarded more highly than others. Football instruction in universities and colleges could be necessary to cross a few of the contextual barriers between machine education and better education. There are individuals who can do it in a number of methods while apparently retaining the framework, standards, and technicality required of higher education but simultaneously integrating the viewer produced, scattered, but also individualized response seen in machine learning. It is compared for the existing *K*-Means method (92.4%) and provided the best result for our ANN method (99.6%) shown in Table 5.

## 4. Conclusions

People can envision a significant response to artificial intelligence in the future because of all practical applications. On the other hand, the management of sports is a critical consideration for the development of the country. There will be guidelines for every athlete if this is possible. Using an Artificial Neural Network model, the quality of football education is examined in this study. To conduct experimental comparative research, the corresponding evaluation indicators are selected using artificial intelligence based on the weight of the indicators. Students' football skills can be improved by fully adopting the AI-based college football practice teaching system model to teach football, according to the findings of the study. Goal and result orientation are also important aspects of football training with AI, as is the ability to implement procedural learning and provide useful feedback. Comparison of the suggested model with the current *K*-Mean method shows that the proposed model has an accuracy rate of 99.6 per cent.

## Figures and Tables

**Table 1:** Machine learning in college and university football teaching categorization frequencies for team A along with team B classifications performance.

Football education in colleges and universities	Frequencies of analysis (%)	
	Team A	Team B
View of the risk prediction playing surface	95.75	97.44
View from above	97.63	96.32
View of the fast playing surface	92.11	94.52
Surface view for slow playing	88.56	89.48
Four or more fouls (penalty)	95.44	86.36
In the fast playing medium surface view mode	89.97	92.32
Close-up surface view (or) out of playing	87.85	78.78
Others	98.43	88.32

**Table 2:** Using AI with machine learning, perform a performance analysis to predict the height of a football player's risk.

Parameters	Training statistics for risk prediction	Statistics for risk prediction testing	Football education at colleges and universities	Football instruction in colleges and universities
Count	65.05	65.03	65.03	65.07
Mean	69.83	78.16	89.85	99.45
Std	4.47	4.43	3.66	4.48
Min	79.23	86.57	73.07	89.53
Max	95.25	96.24	98.72	134.58

**Table 3:** ANN Algorithm with result analysis monitoring for football teaching at students using artificial intelligence and machine learning methods.

Parameter	Monitoring for football teaching vertical (max)	Monitoring for football teaching for vertical (max reach)	Monitoring for football teaching vertical
Count	44.01	46.01	46.03
Mean	38.72	124.06	36.36
Std	3.74	4.9	4.56
Min	39.01	146.51	35.53
Max	48.03	157.04	37.55

**Table 4:** Precision in football teaching using machine learning technique results ANN algorithm using monitoring for ground truth and recall.

Parameters	Ground truth	Recall (%)	Precision (%)
Count	66.03	47.03	53.04
Mean	9.06	19.28	5.37
Std	4.44	2.51	3.12
Min	2.08	25.28	6.18

**Table 5:** Evaluation of football teaching at colleges and universities using machine learning method.

Algorithm	Football teaching in ground truth	Evaluation of the recall value (%)	Evaluation of the precision value (%)
Existing method: *K*-means	31	93.5	92.4
Artificial neural network method	52	95.7	99.6

## Data Availability

The data used to support the ﬁndings of this study are available from the corresponding author upon request.
